# Tobacco plant as possible biomonitoring tool of red mud dust fallout and increased natural radioactivity

**DOI:** 10.1016/j.heliyon.2020.e03455

**Published:** 2020-03-07

**Authors:** Tibor Kovács, Mária Horváth, Anita Csordás, Gergő Bátor, Edit Tóth-Bodrogi

**Affiliations:** a Institute of Radiochemistry and Radioecology, University of Pannonia, 10 Egyetem str., H-8200, Veszprém, Hungary; b Social Organisation for Radioecological Cleanliness, 7/a József Attila str., H-8200, Veszprém, Hungary

**Keywords:** Red mud, Biomonitoring, Tobacco, Po-210, Naturally occurring radioactive materials, Analytical chemistry, Environmental analysis, Environmental chemistry, Environmental pollution, Environmental risk assessment, Natural hazard, Plant biology

## Abstract

Exposure to toxic heavy metal content in soil and inherent naturally occurring radioactive materials (NORM) needs to be monitored, especially after industrial accidents and remediation efforts. Just such an accident happened near Ajka city in Hungary; a large quantity of red mud flooded out from a reservoir. The afflicted area was remediated, and the red mud deposition technology was changed from a wet to a dry procedure. Concerns have been raised about potential hazards from airborne NORM dust in this area. The objectives of this study were to assess the use of explanted tobacco plants as an active biomonitoring system for airborne NORM dust and to reveal weather-related correlations of Po-210 in airborne dust. In 2011, 2012 and 2014, measurements were taken of the following at six monitoring sites in the polluted area and at eight sites in unpolluted areas: soil and tobacco plant Po-210 isotope levels, airborne Rn-222, Ra-226 in soil, Th-232 and K-40 radioactivity concentrations. The transfer factors (TFs) of tobacco were calculated yearly for these isotopes. Association of data with local weather features was determined. In 2012 (the windiest and driest year), the mean Po-210 activity concentrations of tobacco samples in polluted areas were significantly higher than in 2011 and in 2014 (p = 0.044 and p = 0.024, respectively). The mean TF of samples in 2012 was also significantly higher in tobacco plants grown in the polluted area compared to ones grown in unpolluted areas (p = 0.020). These results presumably originate from red mud dust-particle adsorption on tobacco plant leaves. Tobacco plants are promising active bioindicators of airborne particulate pollution by Po-210 or other atmospheric NORM content.

## 1 Introduction

In order to ensure sustainable environmental and living conditions for human society, air and soil quality needs to be maintained (Laville et al., 2006; Morvan et al., 2008; Lehndorff and Schwark, 2010). Assessment of environment qualities, defined as the ability of key environmental constituents such as soil or air to deliver ecosystem services in a sustainable way (Doran and Parkin, 1994), has become a major research topic during the last two decades (Gómez-Baggethun and Ruiz-Pérez, 2011). Toxicant content is one of the most important factors in degradation of these ecosystem qualities and the associated risk of harm to humans (Järup, 2003; Jin et al., 2019). One example is heavy metal pollution. Increasing pollutant metal concentrations augment the potential mobile fraction (PMF) of these metals in soil, depending on their redox reactions and/or acidic solubility (Shaheen et al., 2017). Increased metal PMF will then be the source of system-wide environmental toxicity. In a similar manner, the increased mobility of air pollutants represents a major and systematic environmental risk. Systemic environmental risk, in turn (and without exception), leads to hazards for human lives.

To assess this type of health risk, proper environmental monitoring is necessary worldwide (Wang et al., 2019). If the concentrations of any pollutants increase above maximum allowable values, interventions are required (Antoniadis et al., 2019). For example, bioremediation is an appropriate way of mitigating harmful sources (Zia ur Rehman et al., 2017).

There is a certain amount of overlap between sources and routes of heavy metal pollution and those of naturally occurring radioactive materials (NORM) from anthropogenic sources. Several NORM isotopes have similar geobiochemical cycles to heavy metals (Shotyk et al., 2015). NORM isotopes are Pb-210, Po-210, Rn-222, Th-232, Ra-226 and K-40. Among these, Po-210 represents the most biologically important radiation hazard, partly because of its extreme radiotoxicity and partly because of the ease with which it spreads both in soil and by air. Po-210 and its parent, Pb-210, are soluble in groundwater (or weak acidic media). If ingested or inhaled, Po-210 greatly increases the risk of cancer (Jobbágy et al., 2010), and a high proportion of Po-210 levels is attributable to atmospheric fallout due to the decay of Rn-222 gas. Secondly, another common feature of the cycle of Pb-210 and other Pb-isotopes is their primarily atmospheric deposition. As Po-210 is considered to be the most toxic of all NORM isotopes, proper monitoring of environmental Po-210 levels is required, especially if increased air or soil exposure is supposed (Máté et al., 2013). Biomonitoring of environmental discharge is a favoured option both in industrial risk estimation and after environmental disasters. By using biomonitoring, the level of many potentially harmful sources can be examined at the same time (Burger, 2007) in a representative but relatively economical manner (Nørregaard et al., 2018). Biomonitoring is also an efficient way to monitor the ecological consequences of harmful incidents (Bolsunovsky, 2004) and is capable of detecting the efficiency of remediation efforts, too. By “biomonitoring”, we, therefore, mean the follow-up quantitation measurements of contaminant concentrations or the estimation of toxicant effects in the environment using live organisms (Conti and Cecchetti, 2001; Ernst, 2003; Markert, 2007).

When it comes to combined heavy metal and NORM burden, discharges of municipal and industrial waste, combustion power plants and from the commercial industrial sector (Panagos et al., 2013) can all release both toxicants. As a prime example, agricultural phosphate fertilizer industries discharge both NORM and heavy metals in high enough quantities to increase environmental risks. Inherent NORM release from fertilizer industries may also directly jeopardize the population (Sabiha-Javied et al., 2010) both by air and soil.

Apart from “regular” industrial activities, the same increase in NORM risk evidently applies to large-scale environmental pollution incidents. Such a disaster of large proportions could be exemplified by red mud or red sludge flooding. Not only can this increase toxic heavy metal burden (Wang et al., 2019), but the flooding also leads to an increase in NORM concentrations in the afflicted area (Vandenhove et al., 2009). Red mud flooding has, unfortunately, occurred worldwide from industrial waste deposits, with very severe consequences for the environment. The red mud disasters of Ajka, Hungary (2010), Bento Rodrigues, Brazil (2015) and Brumadinho, Brazil (2019) will have very long-term consequences and present increased hazard risks both in neighbouring communities and the environment.

There is, therefore, a need for biomonitoring methods to be established in environmental remediation with regard to high-risk pollution hazards such as Po-210 NORM increase (Bolsunovsky, 2004). These methods should be easy to deploy in the field, and they should be reproducible and easy to standardize. They should also not require significant amounts of human interaction and should minimize costs. An overview of the literature reveals that in recent decades, terrestrial plant toxicant concentrations have been measured as bioindicators of heavy metal toxicity (Wolterbeek and Bode, 1995; Zheng et al., 2018) in the ecotoxicology of complex systems (Goodman and Roberts, 1971; Markert et al., 2008; Lehndorff and Schwark, 2010; Shaheen et al., 2017). The heavy metal levels of different plant cultures have also been used as bioindicators using vegetable (Zheng et al., 2018) and fruit cultures (El Hayek et al., 2015; Antoniadis et al., 2019). Tree bark (Birke et al., 2018) and tree leaves from various species such as sweet chestnut (Martin et al., 2009), *Acacia sp*. (Attahiru et al., 2015) and *Nerium oleander* (Martín et al., 2018), in their natural habitat, have reportedly been used for heavy metal pollution bioindication.

Reviews and reports indicate the use of mosses (Ares et al., 2012) for lead and 210Pb (Galhardi et al., 2017; Kosior et al., 2017) as NORM retention indicators in ecosystems (Reszka and Wołoszyn, 2007; Shotyk et al., 2015). Grodzinskaya recently published an extensive overview of tree NORM and heavy metal levels in city parks and streets in Kyiv, Ukraine (Grodzinskaya et al., 2019). However, all of the mentioned approaches and reports lack a systematic, engineered, mechanistic and reproducible outplanting of any plant as part of the biomonitoring system. Plant species that are already growing locally are difficult to deploy in a standardized biomonitoring programme in industrial environments, especially trees with long growth periods. In 2003, Ernst introduced the notion of a bioindicator “test plant” (Ernst, 2003), while Markert, in his seminal works, has detailed notions and principles of bioindication and the use of biomonitoring methods with, among others, plant species (Markert, 2007; Markert et al., 2008). Such test-plant reports, however, are still quite scarce, with the notable exemption being a Brazilian report. In this particular study, Silva and co-workers set up an active biomonitoring programme in the vicinity of an oil power plant, with systematically outplanted guava seedlings at different sites and distances (Silva et al., 2013) from the power plant. They successfully determined the risks of increased ozone release and related environmental damage using guava seedlings as a test plant. In another study by Zheng et al. (reported in a Chinese botanics journal), an outplanted bromelid epiphytic “air plant” (*Tillandsia brachycaulos*) was used to detect Pb metal risks (Zheng et al., 2013). As this widely grown, small tropical plant effectively accumulates lead from its primary source (i.e., air fallout), the study provides a good example of a well-deployable active biomonitoring test plant. The same plants are also of use in NORM risk assessment, and Rn-222 removal experiments (Li et al., 2018) have also been based on their exclusive air-breathing leaf systems. In a different approach, site-collected samples of local (not outplanted) nettles (*Urtica dioica*) were used in the study by Olszewski to measure NORM burden near a large phosphogypsum depot (Olszewski et al., 2016).

An industrial site biomonitoring programme can be appropriately established using large-foliage test plants, as it is important to assess both air-related and soil-related risks (Goodman and Roberts, 1971; Ernst, 2003). The use of cultivated large-leaf-surface plants is advisable in this regard, especially given the high importance of atmospheric spread in heavy metal and NORM cycles (Galhardi et al., 2017; Shahid et al., 2017). Tobacco (*Nicotiana tabacum*) fulfils all the necessary criteria for use in an optimal biomonitoring test-plant system. The high environmental tolerance and, uniquely, the known genome of the tobacco plant present additional possibilities for further development in biomonitoring. Importantly, tobacco, with its large and hairy foliage, can account for both air-related and soil-related toxicant risk estimation (Goodman and Roberts, 1971; Papastefanou, 2009). In this regard, its use can be compared to that of nettles but in an easier, outplanted and standardized approach. Our previously published data from earlier validation experiments indicate the plant's applicability in NORM biomonitoring. In the vicinity of a remediated uranium mine, the Po-210 concentration of soil samples and activity concentration of tobacco samples were found to change proportionally (Máté et al., 2013, Máté et al., 2011). Moreover, with eventual low Po-210 concentrations in soil, the characteristic curve is quasi-linear with the Po-210 concentration in the plant's leaves (Máté et al., 2013).

In the study being presented here, the possibility of using tobacco plants for NORM-risk biomonitoring was examined in order to review the bioremediation of a red mud industrial disaster. The northwestern earthen wall of one industrial red mud reservoir near the city of Ajka (Hungary) burst on the 4^th^ of October 2010. Around 700,000 m^3^ of red mud flooded an area of ~10 km^2^, leading to nine deaths and ca. a hundred burn-like personal injuries caused by the sludge's alkalinity (Enserink, 2010). Red mud is an alkalic insoluble residue produced by the aluminium refining industry. It is relatively toxic and poses a serious pollution hazard due to its alkalinity, salt and metal content. It also has an elevated concentration of NORM (Hind et al., 1999; OSSKI, 2010 (National Public Health and Medical Officer Service of Hungary, Sugarbiological Research Institute)).

Before the red mud disaster, wet red-mud-deposition technology was applied in the industrial deposition area, and the slight rise in heavy metal concentrations detected in the vicinity was considered not to be at a toxic level (Juhász et al., 2005; Enserink, 2010). However, in early 2011, after the incident, the red-mud-deposition technology was switched to dry technology, which reduced the moisture content of the red mud (Szépvölgyi and Kótai, 2012). Concerns have, therefore, been raised about inherent radioactivity hazards associated with the NORM content of flying mud dust from both deposits and remediated territories, which was drying out. High NORM-containing particulate matter could become airborne, especially in windy and dry weather conditions. Furthermore, the potential soil/water-borne radioactivity risks of the soil pollutants in red mud remain an open question (Enserink, 2010).

The main objective herein was to determine Po-210 airborne dust hazard by monitoring tobacco plant leaves, soil Po-210 activity concentrations and transfer factors (TFs) in relation to regional weather conditions (wind speed, precipitation and temperature). A secondary goal was to determine activity concentrations of Rn-222 in the air as well as Ra-226 and Th-232 in the soil in order to elucidate possible correlations between the data. Furthermore, K-40 content of red mud could also increase background radiation in the area (Landsberger et al., 2017). Thus, K-40 activity concentrations were also measured in the samples taken over a three-year period.

## 2 Materials and methods

### 2.1 Sampling and sample preparation

**Figure 1:**
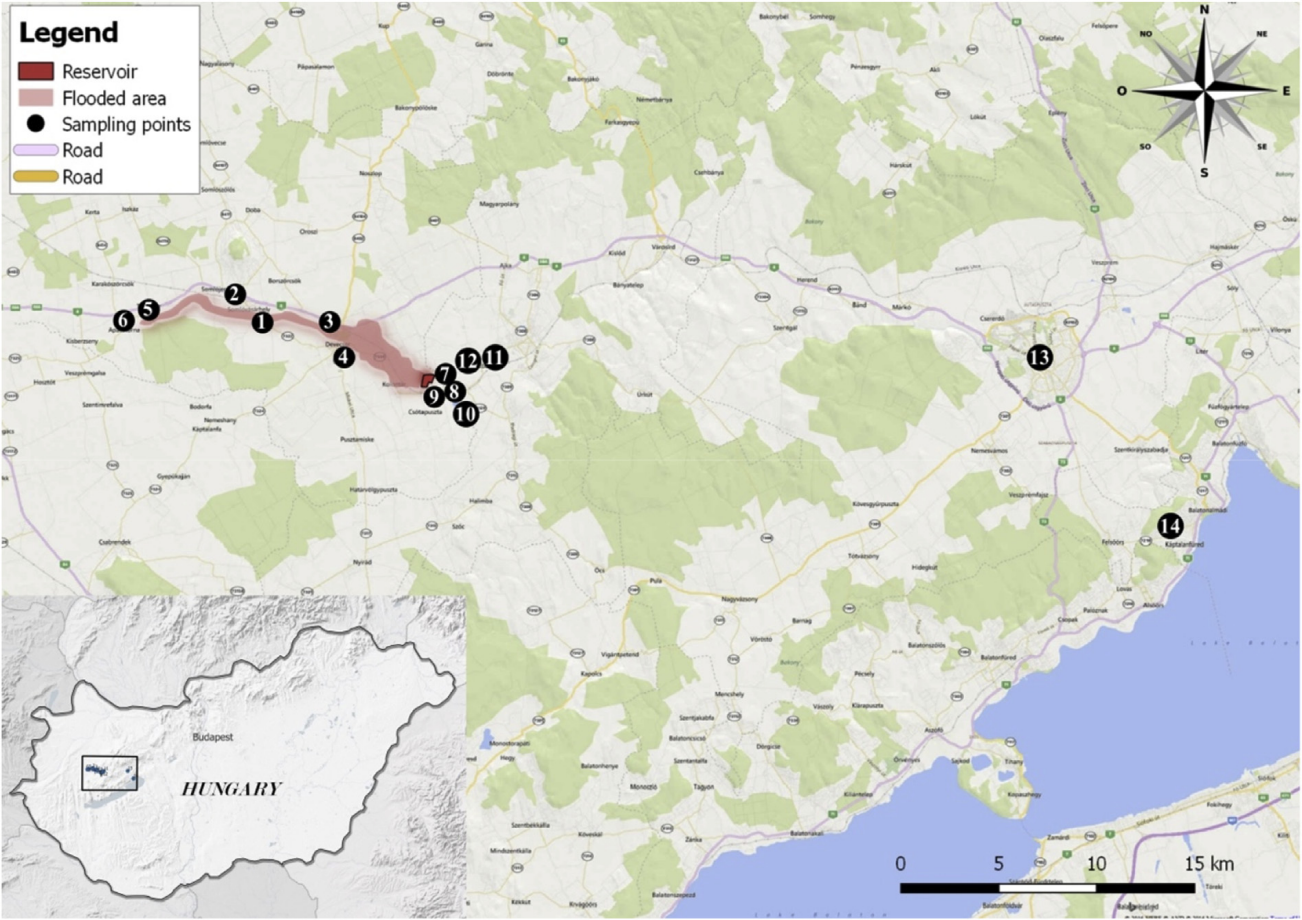
Map overview of the red-mud-flooded area, the deposition and the monitoring sites.

In order to test the biomonitoring function hypothesis, six sampling points (sites), numbered as 1, 3, 5, 7, 8 and 9, were located in the polluted area, while eight sampling points (2, 4, 6, 10, 11, 12, 13 and 14) were also established in unpolluted areas nearby (Figure 1). At each measurement site (n = 4), four tobacco plants were planted. Po-210 activity concentrations were determined in leaf samples (n = 5 leaves from each plant). Thus, per site, twenty (n = 20) tobacco leaf samples were collected and measured for Po-210 activity concentrations (in triplicate). Po-210 activity concentrations in soil samples were also measured, as well as Ra-226, Th-232 and K-40 isotope activity concentrations. To carry out these measurements, three kilograms of soil (in triplicate) were collected per site from the root zone of each plant. This produced a total of twenty soil samples from each of the fourteen sites. Furthermore, in technically feasible site arrangements, Rn-222 activity concentrations in the air were also measured. All samples were collected in the years 2011, 2012 and 2014. Plants were examined during their vegetational periods; they were outplanted on the 15^th^ April and harvested on the 15^th^ September in all examined years. All harvested and dried plant samples were stored for a year at room temperature with 50% moisture content in order to reach Pb-210/Po-210 secular equilibrium (see further details in the Supplementary Information (SI) file).

Weather data were provided by the Hungarian Meteorological Service (HMS) and official local measurement station (Szentkirályszabadja Station). Temperature, wind speed and precipitation-level data were collected with hourly granularity for all days of all six-month vegetational periods in 2011, 2012 and 2014.

In total, Po-210 activity-level measurements were evaluated for all 14 sites using samples from plant leaves and soil, while other soil-based NORM isotope measurements were available for evaluation from a total number of nine sites. Rn-222 air measurement was performed at site numbers 1, 3, 8 and 9 in polluted areas, and at site numbers 4, 11 and 12 in unpolluted areas.

### 2.2 Po-210/Pb-210 measurements

Since the secular equilibrium between Po-210 and Pb-210 is reached in 438 days, the freshly collected tobacco and soil samples were measured after a year of storage (Máté et al., 2013). Consequently, Po-210 activity concentration measurements enable further use of results for Pb-210 determination, too. Details of the Po-210 measurements were given earlier (Máté et al., 2011; Kovács et al., 2007) and are also reported in the SI file of this manuscript.

Po-210 measurements were performed using an Ortec Semi-Conducting Soloist Alpha-spectrometer with a Passivated Implanted Planar Silicon (PIPS) Detector (Ortec, USA). The measurement's duration was 80,000 s, while the minimum detectable activity (MDA) was 0.86–1.91 mBq.

### 2.3 Rn measurements

Rn-222 concentrations in the air were measured by CR-39 track detectors (University of Pannonia, Hungary), with the detectors placed at a height of 0.6 m in Rn-permeable plastic bags. During evaluation, the track detectors were etched (Fábián et al., 2014). They were then assessed with a self-developed image-scanner-based evaluation system (Bátor et al., 2015), and Rn-222 originating signals were counted.

### 2.4 Gamma spectrometry of Ra-226, Th-232 and K-40 radionuclide levels

The Ra-226 activity concentration of soil samples was determined via the radon progenies Pb-214 (295 keV) and Bi-214 (609 keV). Th-232 content was determined from Ac-228 (911 keV) and Tl-208 (2614 keV), and K-40 content was measured with the 1460 keV gamma line (Shakhashiro et al., 2012) using an Ortec GMX40-76 High-Purity Germanium (HPGe) Detector (Ortec, US). The measuring time was 80000 s. For technical (storage) reasons, only the 2014 values were evaluated and are presented.

### 2.5 Statistical methods

The Student's one- and two-tailed t-tests with Bonferroni corrections for multiple comparisons were applied as well as Pearson's correlation tests using SPSS 5.0 (Statistica, US) and Microsoft Excel. Null hypotheses were discarded at p < 0.05. In the case of Pearson r-values, a weak correlation was considered at 0.2 ≤ |r| ≤ 0.4; a moderate correlation was assessed with 0.4 ≤ |r| ≤ 0.7; and a strong correlation was hypothesized at 0.7 ≤ |r|.

## 3 Results and discussion

**Figure 2:**
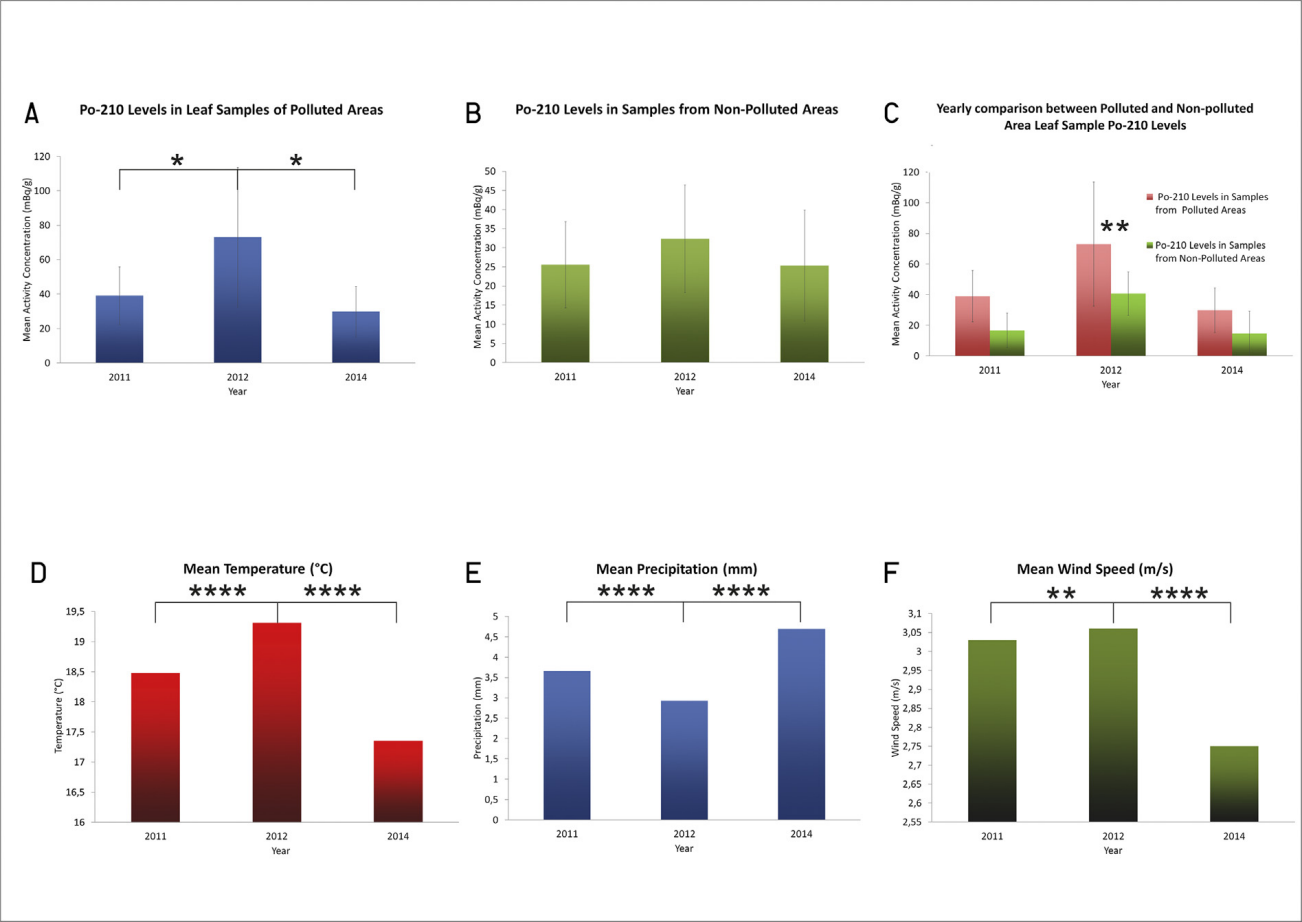
In Panel A, a comparison is made of mean Po-210 activity concentrations in tobacco leaves from polluted and non-polluted sites in 2011, 2012 and 2014. For a comparison of regional weather influence during vegetational periods, yearly mean values of temperature, precipitation and wind speed are shown in Panels B, C and D, respectively. Asterisks denote significance levels: *p* < 0.05 (∗); *p* < 0.01 (∗∗); and *p* < 0.00001 (∗∗∗∗).

In Table 1, the mean Po-210 activity concentration values and their distance from the pollution source (red mud reservoir) are indicated. According to the corresponding Pearson statistical analyses, we could assume that the surfaces of tobacco plant leaves located closer to the reservoir would have collected more dust, hence the increase in Po-210 levels of the measured samples. Figure 2 presents a comparison of mean Po-210 activities measured in each year (both in polluted and unpolluted areas), and it also shows the parallel data for weather conditions. Due to the technological changes and dry weather, there was a higher level of dust fallout in 2012, which increased Po-210 activity concentrations in the soil, too (in comparison with the data for 2011). In 2014, Po-210 activity concentrations in soil continued to increase in areas close to the reservoir. This could be explained by the remedial actions that were subsequently implemented, when all contaminated soil was transported to the reservoir.

**Table 1:** Mean and standard deviation values of radioactivity concentrations in plant leaves (n = 5 leaves from n = 4 plants per site) and soil (n = 3 samples per site) samples at each site. Distance from the red mud reservoir to the sampling site is also indicated.

			2011		2012		2014	
Site number	Polluted (Y)/Non-polluted (N)	Distance (km)	plant (mBq/g)	soil (mBq/g)	plant (mBq/g)	soil (mBq/g)	plant (mBq/g)	soil (mBq/g)
1	Y	12.00	42.30 ± 1.20	42.8 ± 2.80	43.63 ± 2.45	43.21 ± 2.77	17.4 ± 1.69	82.00 ± 5.45
2	N	14.00	27.20 ± 2.66	40.6 ± 3.48	41.67 ± 2.55	47.23 ± 2.39	12.5 ± 1.37	38.80 ± 3.71
3	Y	9.00	7.50 ± 1.01	48.70 ± 2.50	49.50 ± 4.10	47.72 ± 3.68	27.2 ± 1.93	40.60 ± 3.48
4	N	7.20	27.20 ± 2.66	40.60 ± 3.48	34.50 ± 2.38	40.32 ± 2.27	12.5 ± 1.86	38.80 ± 3.72
5	N	19.00	36.25 ± 3.22	33.89 ± 3.40	25.66 ± 2.13	26.60 ± 2.06	9.39 ± 0.96	36.30 ± 2.67
6	N	20.50	20.06 ± 1.25	131.91 ± 9.17	29.60 ± 1.39	125.50 ± 5.34	8.38 ± 1.08	105.40 ± 7.12
7	Y	0.05	45.00 ± 1.02	45.60 ± 1.20	92.10 ± 4.53	102.31 ± 1.51	42.50 ± 1.03	160.60 ± 10.20
8	Y	0.10	46.80 ± 1.00	56.20 ± 1.40	92.50 ± 5.60	102.51 ± 2.11	38.90 ± 1.05	133.40 ± 10.58
9	Y	0.20	56.30 ± 1.60	45.60 ± 2.10	135.00 ± 7.37	76.22 ± 3.81	43.50 ± 1.50	36.40 ± 2.83
10	Y	1.00	43.20 ± 2.30	40.10 ± 2.30	48.50 ± 3.16	45.52 ± 2.37	43.50 ± 2.10	44.20 ± 1.30
11	N	4.00	12.36 ± 1.26	59.60 ± 1.20	9.90 ± 0.88	69.21 ± 5.03	18.30 ± 6.01	64.20 ± 5.07
12	Y	3.50	29.30 ± 2.02	89.20 ± 2.30	39.30 ± 2.30	134.51 ± 5.85	42.30 ± 2.30	82.40 ± 2.50
13	N	38.00	12.76 ± 1.48	53.25 ± 3.82	16.60 ± 1.84	104.21 ± 7.68	18.60 ± 1.36	59.50 ± 2.30
14	N	50.00	13.53 ± 1.56	38.95 ± 3.18	13.50 ± 1.56	28.70 ± 1.82	22.60 ± 2.50	33.50 ± 2.10

### 3.1 Determination of local factors at sites 6 and 11

Sampling site 6 (supposedly an unpolluted site) presents an exception; high soil Po-210 activity concentrations could be measured every year (see Table 1, row 6). This increased Po-210 activity concentration value is a source of bias, i.e., during data review, it was revealed that some years ago, a pottery factory worked onsite. The pottery and ceramics industry is known to increase environmental NORM discharge into soil (Attallah and Abdel-Monem, 2014).

In the case of sample point 11 (an unpolluted site), Po-210 activity concentrations in the tobacco leaves were constantly even lower than in other unpolluted areas. This could be explained by the location of this sampling point. The site is surrounded by a small forest, which absorbs a significant part of all dust. This could cause low Po-210 activity concentrations in plant samples from site 11.

### 3.2 Wind direction as a local influence at site 9

From the available regional meteorological data, it was calculated that the mean degree value of wind direction in the 2012 vegetational season was 290.00°, meaning wind blowing from west-northwest. In contrast, in 2011, the mean wind direction was 243.31°, and in 2014, it was 239.90°. This means that in both other years, wind blew from the southwest to the northeast. (In this meteorological reporting system, 360° = 0° means wind blowing from the north.) At site number 9, in 2012, the Po-210 activity concentrations in plant samples increased, presumably related to the site location being southeast of the reservoir, while the main wind direction in this area also went from west-northwest towards the east-southeast in 2012 only.

This figure (Figure 2 A) presents the tobacco leaf mean Po-210 activity concentrations of polluted versus non-polluted areas for each year examined. Panels B, C and D of Figure 2 also show regional mean temperatures, mean precipitation in mm and mean wind speed values, respectively, in the region during the vegetational period for each year studied.

In 2012, the mean Po-210 activity concentrations of tobacco plants in polluted area samples were significantly higher than in 2011 and in 2014 (p = 0.044 and p = 0.024, respectively). However, in tobacco leaf samples from unpolluted areas, no significant difference in Po-210 activity concentrations was established among the years studied (Figure 2A.). In Table 2, the mean and standard deviation values of radioactivity concentrations are listed for both the polluted and non-polluted area samples of tobacco leaves and soil.

**Table 2:** Plant and soil Po-210 radioactivity means ± standard deviations in tobacco plant samples from the polluted and unpolluted sites monitored in the years 2011, 2012 and 2014. Data are reported without sites 6 and 11 due to local confounding factors revealed during data revision.

Years:	2011		2012		2014	
	Plant (mBq/g)	Soil (mBq/g)	Plant (mBq/g)	Soil (mBq/g)	Plant (mBq/g)	Soil (mBq/g)
polluted area	39.0 ± 16.80	45.50 ± 7.30	73.10 ± 40.60	66.40 ± 32.10	29.80 ± 14.20	81.60 ± 54.20
unpolluted area	25.53 ± 11.30	50.45 ± 19.71	32.34 ± 14.16	66.73 ± 42.35	25.32 ± 14.15	49.53 ± 18.41

This set of data clearly indicates that among the years 2011, 2012 and 2014, 2012 was the hottest (with the highest mean temperature) and driest (with the lowest precipitation values) during the vegetational period; p-values for compared means (2012 vs. 2011 and 2012 vs. 2014) are in the range 0.00001 or below (see Figures 2 B, C and D).

**Figure 3:**
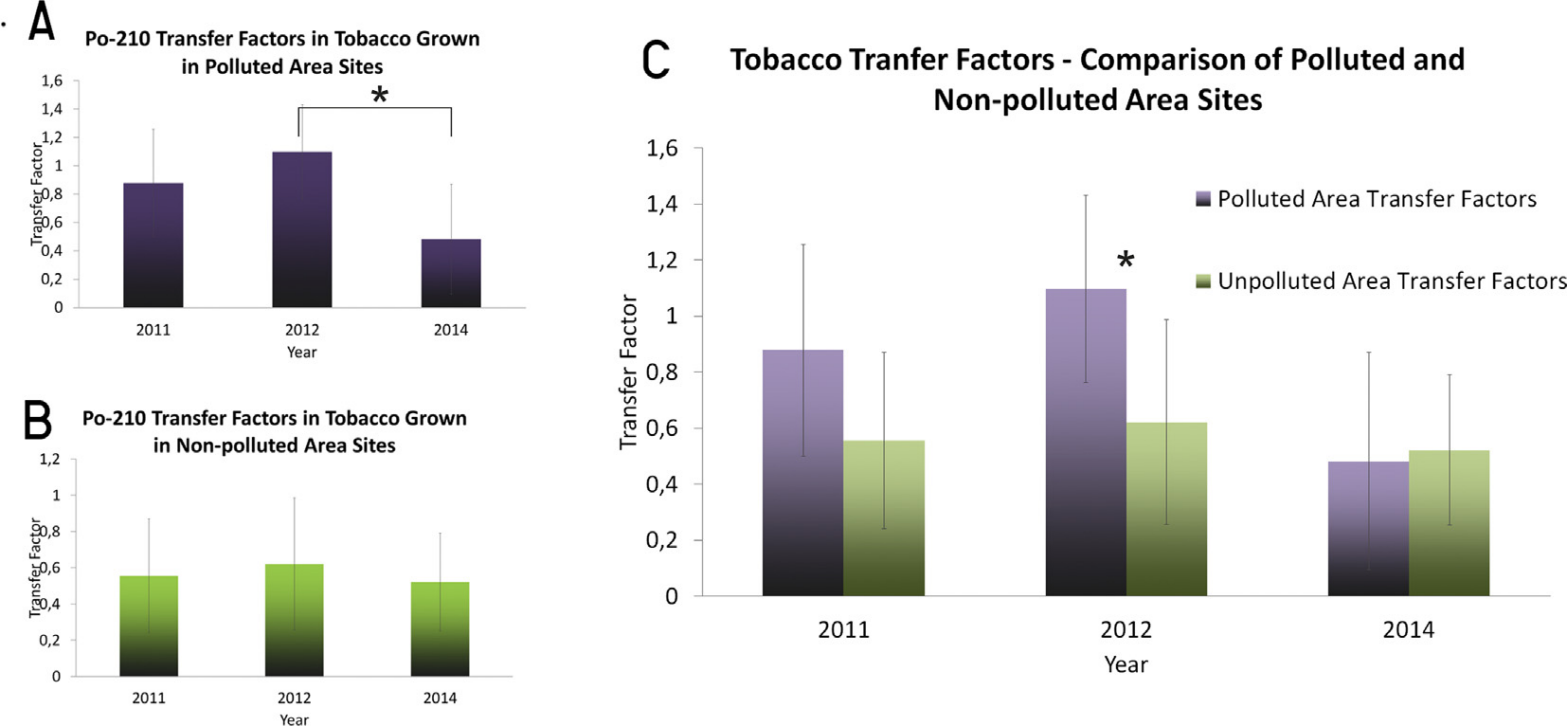
Calculated yearly mean Po-210 transfer-factor values for tobacco plant leaves in polluted and unpolluted areas. Yearly differences and differences between polluted and non-polluted samples are denoted with an asterisk, meaning *p* < 0.05.

TFs are calculated as a ratio of specific metal concentrations in plant tissue to the concentration of the same metal in soil (Mirecki et al., 2015). Mean TFs are shown in Table 3. In 2011, 2012, mean TFs were significantly higher in polluted samples than in the unpolluted samples for the same year (p = 0.022 and p = 0.005 for 2011 and 2012, respectively) (see Figure 3.). To avoid the previously mentioned distorting effects of soil pollution derived from earlier ceramics production and decreased plant activity concentrations due to the local forest belt, results were calculated without data from sites 6 and 11. As the effect of wind and dustability was a focus in this study, data from sample point 9 were still included in our analysis. However, for the sake of completeness, both types of data are featured in Table 3.

**Table 3:** Transfer factor mean values and standard deviations of tobacco plants for each year at polluted and unpolluted sites.

Transfer Factors (mean ± standard deviation)	2011	2012	2014
polluted area	0.88 ± 0.38	1.10 ± 0.33	0.48 ± 0.39
unpolluted area without confounding data of Site 6, 11	0.55 ± 0.31	0.62 ± 0.36	0.52 ± 0.27
unpolluted area with data from Site 6 and 11 included	0.46 ± 0.32	0.51 ± 0.37	0.43 ± 0.28

In 2011, 2012 and 2014, the mean calculated TFs were 0.55 ± 0.31, 0.62 ± 0.36 and 0.52 ± 0.27, respectively. However, even with bias correction, significant differences can still be seen between polluted and unpolluted sample TFs in 2012 (p = 0.020), supporting the conclusion that the tobacco plants detected increased NORM dust fallout.

### 3.3 Results for the correlation between distance from the pollution source and tobacco leaf Po-210 levels

In terms of the correlation between the source distance and radioactive concentrations measured in tobacco leaves, the Pearson r-value was found to be -0.34 for the results of 2011, -0.57 for 2012 and -0.85 for 2014. Thus, in 2011, there was a weak negative correlation, while in 2012, there was a moderate negative correlation. In 2014, however, there was a strong negative correlation between distance from the source and Po-210 radioactive concentrations in tobacco leaves at each individual site. Results (presented in Table 1) and the corresponding Pearson statistical analyses indicate that the surfaces of tobacco plant leaves located closer to the reservoir could have collected more dust, hence the increased Po-210 activity in measured samples. Meteorological data on the most humid year (2014) indicate that decreasing airborne PM levels could be the most prominent reason for the strong negative correlation between the distance of plants from the discharge source and their Po-210 radioactivity concentrations.

The Pearson r-value was -0.40 for soil radioactivity results in 2011, -0,67 for 2012 and -0.49 for 2014. While correlation coefficients relating to soil (or rather, dust fallout to soil) radioactivity show a clear trend (and effect) of humidity and hence that distance dependence increases with increasing dustability and increased fallout by drier weather conditions, the actual correlation factors describe a moderately negative correlation between soil radioactivity and distance from the radioactivity pollution source.

Po-210 (and Pb-210 as well) has a high migration rate in a weak acidic medium (Vandenhove et al., 2009). This points to increased groundwater transportation with increased humidity, but in the light of previous investigations (Persson and Holm, 2011), as a Pb-decay product, Po-210 pollution could bypass soil migration with airborne Po-210 dust, transported further with wind and rainwater fallout. Atmospheric deposition of heavy metals is possible and, in the case of lead isotopes, is actually the most prominent means of spreading (Persson and Holm, 2011; El Hayek et al., 2015; Birke et al., 2018, Jin et al., 2019). Wind was the strongest in 2012, and the highest number of windy days also occurred in 2012. The weakest wind speeds but highest levels of precipitation in the region were recorded in 2014; it is plausible that the correlations of Po-210 levels are associated with the windy 2012 and humid 2014 vegetational period. Transfer factors represent proportional Po-210 content in the plant to that of the soil in which it grows (Mirecki et al., 2015). It is remarkable that significantly higher transfer factors were measured in the driest year (2012) than in the most humid year (2014). Similarly, the difference in transfer factors for tobacco plants in polluted areas was significant between 2012 and 2014. In the more humid yearly vegetational period of 2014, significantly lower TFs were obtained compared to the driest year of 2012. This, therefore, points to another indication that leafy plants are a sensible choice for outplanted biomonitoring of the complex environmental spread of several toxicants.

### 3.4 Rn concentration ranges

Rn concentration was found to be between 9.32 and 92.06 Bq/m^3^ in 2012, and between 12.49 and 133.41 Bq/m^3^ in 2014 at a height of 0.6 m. The global annual average Rn concentration in the air, when measured outdoors, is 5–10 Bq/m^3^.

Due to changes in technology as well as dry weather turning the red mud into dust, the relationship between increased Po-210 activity concentrations on leaf surfaces and Rn-222 concentrations in the air was also investigated. The Pearson r-value was -0.43 for the results from 2012 and -0.39 for 2014. This means that only a moderate relationship was found between plants’ Po-210 concentrations and Rn-222 concentrations in the air. This points to atmospheric dust being the most important toxicant delivery system in relation to red-mud-related NORM risks.

### 3.5 Naturally occurring radioactivity concentration levels

Ra-226, Th-232 and K-40 activity concentrations in soil samples were determined with gamma spectrometry for the year 2014, comparing polluted and unpolluted sites. Table 4 contains these results.

**Table 4:** NORM radioactivity concentrations in soil samples from different sites in 2014. Samples are firstly separated with the results of polluted area samples listed in the first five rows and non-polluted area samples in the later rows.

Site Nr.	Polluted (Y)/Non Polluted (N)	Ra-226 [Bq/kg]	Th-232 [Bq/kg]	K-40 [Bq/kg]
1	Y	141.8 ± 48.7	31.4 ± 11.9	402.6 ± 21.5
3	Y	168 ± 58.0	104.9 ± 25.0	399 ± 21.4
7	Y	139.5 ± 48.0	46.5 ± 15.0	379 ± 20.6
12	Y	159.4 ± 55.1	19.6 ± 9.3	306.9 ± 17.9
4	N	55.1 ± 20.0	22.4 ± 9.8	371 ± 20.3
5	N	58.2 ± 25.4	29.1 ± 11.3	479.6 ± 24.3
6∗	N	216.2 ± 74.0	23.6 ± 10.0	469.9 ± 23.9
9	N	36.7 ± 18.0	26.4 ± 10.8	326.9 ± 18.7
11	N	102 ± 31.8	47.1 ± 15.0	672.9 ± 31.1

∗ The unusually high Ra-226 level of the soil at site 6 (non-polluted) is due to existing contamination from NORM-raising ceramics manufacturing.

Comparing Table 1 with other NORM data in Table 4, we see that there were no significant differences in the radiological parameters between soil samples from affected and non-affected regions. The outlier site 6, with its correspondingly high soil Ra-226 level, has already been discussed.

Pearson statistical analyses show an r-value of 0.07 between the Ra-226 content of soil and Rn-222 activity concentrations in the air. Thus, no relationship was found between any soil Ra-226 concentration and Rn-222 concentration in the air. This outcome is in line with expectations because Rn-222 activity concentrations in the air depend on several different parameters, e.g., the type of soil, its composition, porosity and exhalation rate.

Taken together, the tobacco leaf measurement results and TF findings are perfectly in line, indicating a dominant washable, dust-related environmental NORM risk. Consequently, we can emphasize that the role of dustability and dust-borne NORM burden increases available pollution sources during dry years. Data on wind directions and, specifically, the observed effects of dust transportation by wind at site 9 lend further credence to our suggestion of tobacco as a biomonitoring “test plant”. Po-210 activity concentration values in both tobacco plants and the soil of polluted areas are identical to the mean values of samples taken from other areas (except the results for tobacco leaves collected in 2011 and 2012, where increased levels of Po-210 activity concentration values were found). This fact again indicates the role of airborne dust monitoring by the tobacco plant bioindication method.

It was apparent that tobacco plant leaf measurements could track the risk of airborne NORM spread that bypassed soil migration (as lower TFs in the humid 2014 vegetational season also support this hypothesis). Previous studies of plants and mosses, used in bioindication of diverse metal-related toxicants, also show the prominent role of airborne pollution. The importance of monitoring air-related pollution has been shown by Shotyk in Germany (Shotyk et al., 2015) and Kosior in Poland (Kosior et al., 2017). The data and concentration ranges obtained to measure lichen, moss, some higher plant metals and NORM levels (Grodzinskaya et al., 2019; Galhardi et al., 2017; Martín et al., 2018) are in line with the results obtained using our measurement techniques. The ranges of our obtained values are in perfect agreement with other studies reporting Po-210 environmental concentrations (Reszka and Wołoszyn, 2007; Shotyk et al., 2015; Kosior et al., 2017, Galhardi et al., 2017). Soil K-40 ranges are also in line with other measurements, such as those reported by Mehra (Mehra et al., 2007). Thus, the results reported here also indicate a standardization possibility for this test-plant method. Those studies that report leafy plants (Guava, Oleander, Acacia, Nettle, Opuntia, Castanea or water plants) for the estimation of heavy metals or NORM have all successfully detected either airborne effects of pollution or air-pollution-related increases in toxicant concentrations in leaves (Goodman and Roberts, 1971; Bolsunovsky, 2004; Golubev et al., 2005; Martin et al., 2009; Lehndorff and Schwark, 2010; El Hayek et al., 2015; Olszewski et al., 2016; Galhardi et al., 2017). By applying outplanted tobacco test plants, our study has come to a similar conclusion in terms of the potential of leaves for airborne, metal-related toxicity monitoring.

## 4 Conclusions

Data on Po-210 activity concentrations in the leaves of tobacco plants, together with activity concentration data from soil samples, show that Po-210 concentrations in tobacco leaves increased in polluted and remediated areas after red mud flooding. The results imply that the tobacco plant not only absorbs Po-210 through its roots but is also capable of absorbing radionuclides via its leaf surfaces (Berger et al., 1965; Wolterbeek and Bode, 1995; Vandenhove et al., 2009; Shahid et al., 2017). These NORM radioactivity increases can be attributed to the change in technology for red mud depositing/storage in 2011 (from wet technology to a dry procedure), as well as to the 2012 period of dry and windy weather.

Tobacco plants as indicators were found to present annual weather-related and distance-related changes in environmental NORM concentrations in dust. Tobacco plant transfer-factor measurements indicated a bypassing of soil water-borne NORM spreading in dry and hot weather (Lehndorff and Schwark, 2010). In summary, tobacco plants could be used to monitor air-particulate-matter-related pollution, too, besides their already known bioindication role in soil Po-210 pollution (Máté et al., 2011) monitoring. In our survey, other confounding factors and effects of Po-210 pollution from other sources needed to be considered. One such factor was the airborne-radionuclide-level decreasing effect of local forest belts. Thus, representative siting and sample collection are essential for this monitoring method. Moreover, increasing transfer factors may also indicate higher Po-210 airborne dust or increased rain-fallout levels, but to elucidate specific correlations, further studies are required. The results presented here could, therefore, indicate a more prominent role for biomonitoring of NORM increases using tobacco test plants in affected areas in the future. The results of recent reviews and biomonitoring studies also all point to leafy plant use for estimation or tracking airborne pollution.

## Declarations

### Author contribution statement

Tibor Kovács, Edit Tóth-Bodrogi: Conceived and designed the experiments; Analyzed and interpreted the data; Contributed reagents, materials, analysis tools or data; Wrote the paper.

Mária Horváth, Anita Csordás, Gergő Bátor: Performed the experiments; Analyzed and interpreted the data.

### Funding statement

This work was supported by Hungarian National Research OTKA grant No. K128805 and K128818 and GINOP Grant of the Hungarian Government No. 2016-0016.

### Competing interest statement

The authors declare no conflict of interest.

### Additional information

No additional information is available for this paper.

## Appendix A Supplementary data

Supporting_Information_Tobacco_V2 Supporting_Information_Tobacco_V2
