# Supplementary material for: Tobacco plant as possible biomonitoring tool of red mud dust fallout and increased natural radioactivity
Source: Heliyon. 2020 Mar 7;6(3):e03455. doi: 10.1016/j.heliyon.2020.e03455 (PMC7062938; doi:10.1016/j.heliyon.2020.e03455)
Supplement: Supporting_Information_Tobacco_V2 [file mmc1.docx]

***Tobacco plant as possible biomonitoring tool of red mud dust fallout and increased natural radioactivity***

Tibor Kovács^1,2*^ , Mária Horváth^1,2^, Anita Csordás^1^, Gergő Bátor^1,2^, Edit Tóth-Bodrogi^1^

^1^ Institute of Radiochemistry and Radioecology, University of Pannonia, 10 Egyetem str., H-8200, Veszprém, Hungary

^2^ Social Organisation for Radioecological Cleanliness, 7/a József Attila str., H-8200, Veszprém, Hungary

*Corresponding author: Tibor Kovács, PhD;

Telephone: +36 88 624-789, Fax: +36 88 624-178

**Supporting Information**

Po-210 measurements

Additionally to Po-210 measurements, for other aims outside the scope of this report, Pb- 210 levels had to be also measurable. Since tobacco has a growing period of 6 months and the  secular equilibrium between Po-210 and Pb-210 is reached in 438 days, the secular equilibrium between Pb-210 and Po-210 could not set during the tobacco growth. In case of freshly collected samples the equilibrium state of Po-210 and Pb-210 is unknown, therefore further determination of Pb-210 activity concentration via the measurement of Po-210 activity concentration could be performed only after the samples being stored for an appropriate amount of time. In the stored soil or tobacco samples after a year of storage Pb-210 and Po- 210 are in secular equilibrium (Máté et al. 2013). Consequently their Pb-210 activity concentration could be determined via Po-210 activity concentration measurements. Therefore samples were stored to reach secular equilibrium and enable the further use of results for Pb-210 determination, too.

32.96 mBq of Po-209 as recovery tracer was added to 2 g of the sample, which was digested with a combined acidic solution as described earlier (Máté et al. 2011). During the digestion first 25 mL concentrated (cc.) HNO3 was added to the samples in order to digest the organic materials. The solution was evaporated to around 5 mL. This step was repeated three times. After this 25 mL cc. HCl was added to the evaporated solution in the same conditions to elimate disturbing nitrate ions. Five drops of H2O2 were added to it to digest the organic compounds and finally the HCl concentration was reduced with ultrapure destilled water. 100 mL 0.5 M HCl stock solution was prepared from the residue (Kovácset al. 2007). The suitable source for alpha-spectrometry was prepared using spontaneous deposition at 80oC on a stainless steel plate with high nickel content (WNr. 1.4539, DIN 17740, 25% Ni). 100 mg ascorbic acid was added to reduce Fe3+ ions (Jobbágy et al. 2010). The source was dried at room temperature and was measured with the semi-conducting Ortec Soloist alpha-spectrometer with Passivated Implanted Planar Silicon (PIPS) detector (Ortec, USA).

Rn measurements

Rn-222 concentration in the air was measured by CR-39 track detectors (University of Pannonia, Hungary) with the detectors placed at a height of 0.6m in Rn-permeable plastic bags. Rn measurement was performed in sampling sites nr. 1, 3, 8, 9 in polluted areas and in ites nr. 4, 11 and 12 in unpolluted areas. More Rn measurements were not feasible technically at the other sites. During evaluation the track detectors were etched in a 6 M 90 °C NaOH solution for three hours (Fábián et al. 2014). Then they were assessed with a self- developed, scanner-based evaluation system (Bátor et al.2015). During the procedure the detector surface is scanned and converted to a monochrome image. In the image the area of interest is selected and the number of counts is summed. Pictures from reflective and transmissive scans are analysed using in-house developed specific Image Analyzer (IA) software.

Gamma-spectrometry

Gamma-spectrometry of Ra-226, Th-232, K-40 radionuclide levels The Ra-226 activity concentration of soil samples was determined via the radon progenie Pb-214 (295 keV) and Bi-214 (609 keV), the Th-232 content was determined from Ac-228 (911 keV) and Tl-208 (2614 keV) and the K-40 content was measured from the 1460 keV gamma line (Shakhashiroa et al., 2012) using an Ortec GMX40-76 High-purity Germanium (HPGe) detector (Ortec, US). The measuring time was 80 000 s. For technical reasons (storage) only the 2014 values were evaluated and are presented.
